# Desired Support and Design Preferences for a Supported Self‐Management Intervention for People With Lower‐Grade Gliomas: Co‐Design Findings From the Ways Ahead Project

**DOI:** 10.1002/pon.70561

**Published:** 2026-07-27

**Authors:** Ben Rimmer, Sophie Williams, Joanne Lewis, Lizzie Dutton, Richéal Burns, Pamela Gallagher, Vera Araújo‐Soares, Tracy Finch, Linda Sharp

**Affiliations:** ^1^ Population Health Sciences Institute Newcastle University Newcastle University Centre for Cancer Newcastle upon Tyne UK; ^2^ Newcastle upon Tyne Hospitals NHS Foundation Trust Newcastle upon Tyne UK; ^3^ Faculty of Science Atlantic Technological University Sligo Ireland; ^4^ Health and Biomedical Strategic Research Centre Atlantic Technological University Galway Ireland; ^5^ School of Psychology Dublin City University Dublin Ireland; ^6^ Department for Prevention of Cardiovascular and Metabolic Disease, Medical Faculty Mannheim Centre for Preventive Medicine and Digital Health Heidelberg University Heidelberg Germany; ^7^ Department of Nursing, Midwifery and Health Northumbria University Newcastle upon Tyne UK

**Keywords:** brain tumours, cancer, co‐design, intervention, lower‐grade gliomas, oncology, self‐management

## Abstract

**Background:**

Lower‐grade gliomas (LGG) are a subgroup of primary brain tumours. People with LGG often live long‐term with wide‐ranging symptoms and impairments (e.g., seizures, cognitive impairment) and uncertainty about their disease progression. Self‐management interventions can improve quality‐of‐life (QoL) in cancer survivors; however, adaptability, acceptability, and feasibility of existing interventions for people with LGG is unclear.

**Aims:**

Co‐design, with multiple stakeholders, an early prototype supported self‐management intervention for people with LGG.

**Methods:**

Co‐design activities followed two sequential phases. Phase one comprised three semi‐structured interview sets with people with LGG, caregivers, and healthcare professionals (HCP). Desired support and design preferences were inductively identified and mapped to the TIDieR checklist; this informed a paper prototype outline. Phase two comprised four discussion groups with people with brain tumours, caregivers, and HCPs, and a survey. These activities considered the prototype design, feasibility, and acceptability. A preliminary intervention logic model was developed from Phases one and two, evidence and self‐management theory.

**Results:**

Early co‐design findings indicate preference for a blend of online and face‐to‐face access to information and support with consideration given to technology literacy and accessibility. A support ‘toolkit’ was desired for advice, signposting, and support, with the option for caregivers to access intervention content, where appropriate. To improve QoL, the intervention needs to improve symptom knowledge and self‐efficacy for symptom management, and support psychological adjustment and independence.

**Conclusions:**

This groundwork, involving multiple stakeholders, will help ensure intervention acceptability, feasibility and effectiveness. There is scope for wider applicability to other brain tumour groups.

## 1 Background

Lower‐grade gliomas (LGG) are a subgroup of primary brain tumours that are incurable and limit life expectancy [1]. People with LGG experience wide‐ranging symptoms and impairments (e.g., fatigue, seizures, cognitive impairment), that can require long‐term management alongside the uncertainty of an incurable condition [2].

Self‐management is ‘awareness and active participation by the person in their recovery, recuperation, and rehabilitation to minimise the consequences of treatment, promote survival, health and well‐being’ [3]; for example, use of equipment, acquiring knowledge, and self‐monitoring. Social cognition theories suggest self‐management is underpinned by an individual's self‐efficacy [4]. Those closest to people with chronic conditions often become caregivers,^1^ who alongside healthcare professionals (HCP), can help equip people with the skills to self‐manage effectively [5].

Self‐management interventions show promise for improving self‐efficacy, quality‐of‐life (QoL) and reducing healthcare utilisation in cancer survivors [6]. Such improvements are associated with components from the practical reviews in self‐management support (PRISMS) taxonomy [7], particularly ‘Information about the condition and its management’ and ‘Training for psychological strategies’ [6]. However, adaptability of existing interventions to people with LGG is uncertain. Moreover, self‐management interventions have not been widely implemented, indicating a need to address acceptability and feasibility concerns in development [8].

A 2021 Call to Action identified six priority actions to improve integration of self‐management support in cancer care [9]. We aimed to co‐design an evidence‐based and theory‐informed supported self‐management intervention for people with LGG, working with multiple stakeholders to ensure acceptability, feasibility, and applicability to the needs of people with LGG. Here we report early‐stage co‐design activities and findings [10], providing a novel understanding of how supported self‐management for people with LGG might be achieved.

## 2 Methods

This multi‐methods project (‘Ways Ahead’) was approved by the Wales Research Ethics Committee (REC ref: 20/WA/0118). Full details of methods and empirical findings are reported elsewhere (Supporting Information S1: Table 1); the evidence‐base from Ways Ahead established the need for, and informed potential content of, a supported self‐management intervention. Co‐design activities followed two sequential phases (Supporting Information S1: Table 2). To be eligible for either phase, people with LGG and caregivers had to be aged ≥ 18 years and have completed primary treatment for, or have supported someone living with, a grade 2 astrocytoma or grade 2 or 3 oligodendroglioma; HCPs had to be involved in formal care or support of adults with brain tumours.

Phase one comprised three semi‐structured interview sets with people with LGG (*n* = 28), caregivers (*n* = 19), and HCPs (*n* = 25). Recruitment adverts were disseminated to four United Kingdom National Health Service hospitals and the Brain Tumour Charity's research involvement networks. Those who registered interest were purposively selected to maximise diversity in experiences (e.g., age, sex, tumour type, relationship to care recipient, healthcare profession). As well as exploring experiences of LGG (Supporting Information S1: Table 1), two interviewers (BR, PhD; LD, PhD) asked participants what support would help people with LGG engage in self‐management and design preferences. Informed consent was obtained and interviews were audio‐recorded, transcribed, and analysed using the framework method [11]. BR and LD inductively double coded transcripts to identify areas of desired support and design preferences; disagreements were resolved through discussion. BR then deductively mapped codes to the Template for Intervention Description and Replication (TIDieR) checklist of intervention characteristics (e.g., how, who, when, where, what) [12] (Table 1; Supporting Information S1: Table 3). From these findings, the team designed an early paper prototype intervention (Supporting Information S1: Table 4).

**TABLE 1:** Design preferences for a supported self‐management intervention for people with LGG.

Intervention characteristic	Key findings	Illustrative quotes from phase one interviews
What (content)	Wide‐ranging topics (e.g. physical, cognitive, psychological, practical, social, role); support needs vary Outline what to expect at different timepoints (e.g. what might change/long‐term impact); share this with caregivers Support with cognitive deficits and psychological adjustment most important	‘There's support with occupation, finance, psychological, and physical things…even dietary, health and fitness… So it's having access to all these.’—HCP39 (clinical nurse specialist) ‘Help the [support recipient] to understand why they feel frustrated or why they've got no patience anymore and then ways to cope with that and self‐manage. That to me is the essence of self‐management, giving people not just the understanding but the tools to do it for themselves.’—C23 (Male spouse)
What (materials)	Centralised ‘living’ resource with contact details, self‐management advice, and signposting Consider technology literacy and accessibility for online information. Keep information accessible (e.g. level of detail, video/audio/visual information)	‘I think a central depository of information… so if I want to know stuff about diet, somewhere I can call or look up.’—Pa13 (Male, grade 3 oligodendroglioma) ‘We found that people are quite liking the education sessions that are pre‐recorded so just being able to get the information in different ways so that they can access it differently if they want, whether it be sent out in a leaflet or an online video.’—HCP52 (Epilepsy nurse specialist)
Who (if not self‐administered)	No preference, providing support provider(s) are knowledgeable and empathetic HCP referral/signposting legitimises the reputability of information/support	‘Lay people or professionals, a mix, as long as they're competent, they know what they're talking about and they're empathetic.’—Pa16 (Male, grade 3 oligodendroglioma)
How	Groups offer opportunities to signpost, share experiences, and connect with others; but groups not acceptable to all Blended approach of online and face‐to‐face necessary Integrate caregivers in support/information provision	‘I go on the brain tumour support groups for a lot of information…and people can give advice to you of their experiences, which is a good help sometimes.’—Pa30 (Male, grade 3 oligodendroglioma) ‘Immediately I thought, “Families, carers need to be part of this as well,” because I think they can reiterate back to patients what the advice has been or what they've actually learnt.’—HCP49 (Occupational therapist)
Where	Favour community‐based locations for any face‐to‐face support Online support mitigates issues with mobility/transport; but interaction is more difficult in online formats	‘Online support would be good…because a lot of people have mobility issues afterwards.’—C05 (Female spouse) ‘If people have got issues with driving that could be a difficulty as well. So, yeah, I guess [face‐to‐face support] would be good but there might be some practical barriers to overcome.’—Pa10 (Female, grade 2 oligodendroglioma)
When	Needs change over time; information to be available as and when needed Initial needs assessment post‐primary treatment, once had time to adjust	‘A support package put in place for you, and you can dip in and out of that at different times.’—Pa11 (Male, grade 2 oligodendroglioma) ‘For most people who go through treatment, I think it's busy, busy, busy, there's something to work towards for months and months and then all of a sudden it's, “Right okay,” and then often that's where I find people tend to unravel a bit.’—HCP18 (Psychologist)
Tailoring	Identify support needs and prioritise support areas Signpost to information and support relevant to individual needs	‘Identification of issues then developing patient goals of what they want to achieve and how we can support that.’—HCP21 (Physiotherapist) ‘My problems are based around my disability. I Need physical support… some people I know have emotional needs, but others don't… you've got to design it around the individual.’—Pa13 (Male, grade 3 oligodendroglioma)

*Note:* This table is comprehensively expanded in Supporting Information S1: Table 3.

Phase one findings informed phase two, which comprised four sequential discussion groups (two with people with brain tumours, *n* = 14; one with caregivers, *n* = 4; and one with HCPs, *n* = 6) and a survey (of people with brain tumours and caregivers; *n* = 33) (Supporting Information S1: Table 2). To recruit, we used opportunity sampling, and advertised across charity and support networks. Phase two participants included people with any brain tumour type for early examination of whether this work could extend to people with similar symptoms and impairments (e.g., seizures, cognitive impairment), irrespective of tumour type.

BR and LS (PhD) facilitated the groups, which discussed the design, feasibility, and acceptability of the prototype intervention to iteratively develop and refine ideas. The study‐developed survey (Supplementary file) asked people with brain tumours and caregivers open and closed questions about the prototype, structured around the TIDieR checklist [12]. Free‐text responses were cross‐referenced with Phase one findings to further develop understanding of design preferences. Closed question responses provided larger‐scale quantitative understanding of potential acceptability of design features. Phase two findings were used to revise the prototype intervention and inform its guiding principles and preliminary logic model.

## 3 Results

Table 1 (expanded in Supporting Information S1: Table 3) outlines key findings, with interview quotes, for desired support and design preferences, in accordance with the TIDieR checklist [12]. Supporting Information S1: Table 4 presents the refined prototype and guiding principles. In brief, people with LGG (plus caregivers, where desired) will have six one‐monthly remote appointments with a trained advisor to identify support needs and be directed to appropriate information and support within an interactive web‐app.

**FIGURE 1:**
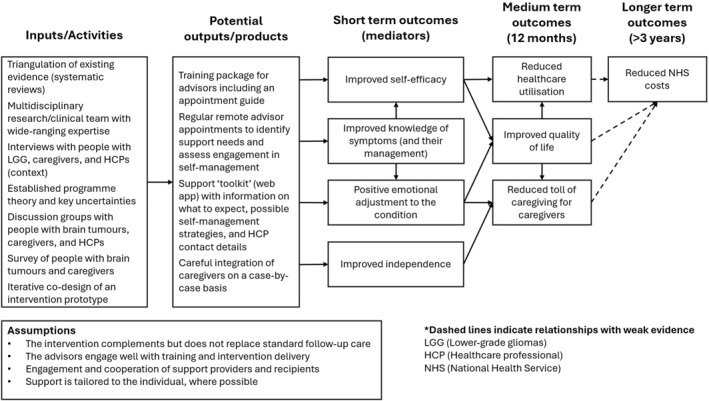
Preliminary logic model of change for the intervention.

The preliminary intervention logic model (Figure 1) outlines relationships between the activities, outputs and possible outcomes. To improve QoL, the intervention needs to improve symptom knowledge and self‐efficacy for symptom management, and support psychological adjustment and independence; this is dependent on support provider and recipient engagement. Below we expand on three areas of desired support where preferences were consistent and strong across participant groups, to highlight acceptability and feasibility considerations.

### 3.1 Support ‘Toolkit’

All interview sets reported desire for a ‘toolkit’ applicable to the needs of people with LGG, encompassing signposting to available support, advice on what to expect, self‐management strategies, and contact details and support roles of HCPs.

Discussion groups highlighted that technology literacy and accessibility was a possible barrier to a digital toolkit, though most survey respondents were willing and confident to use a web‐app/website/app. Participants acknowledged the difficulty with keeping a physical resource up‐to‐date; people with LGG and caregivers emphasised the need to access different supports at different timepoints, underlining desires for a centralised ‘living’ resource. To reduce information overload and improve accessibility, clearly labelled sections with controlled levels of detail and appropriate use of videos/visual information were suggested.

### 3.2 Group Support

People with LGG and HCPs highlighted the value of groups for sharing advice and experiences. However, challenges of availability, location, timing, and (in)sufficient interest were raised. Discussion groups suggested online groups could facilitate attendance, affording flexibility in when to attend; while face‐to‐face groups offer relaxed environments with breakout interactions. A blended approach was judged to offer people the opportunity to decide what is acceptable to them. Focussed discussion topics on specific areas of self‐management and relevant strategies were desired. Survey respondents suggested group support should be available alongside a support ‘toolkit’; though for many people with LGG, meeting ‘similar others’ was a reminder of their incurable condition, often deterring involvement.

### 3.3 Careful Integration of Caregivers in Support

All interview sets valued ‘supported’ self‐management, emphasising key roles for caregivers and HCPs. People with LGG and caregivers stressed a need for caregivers to know what to expect and how they can help. The HCP discussion group acknowledged how caregivers can help implement self‐management strategies in day‐to‐day life. However, an intervention should not rely on caregivers, as support networks vary; instead, caregiver integration should be considered on an individual basis, with focus on encouraging the support recipient's independence.

## 4 Discussion

Possible long‐term management of wide‐ranging symptoms and impairments [2], and the potential for self‐management interventions to improve QoL [6], highlights the need to develop a self‐management intervention for people with LGG. This early, iterative, co‐design work followed the Medical Research Council guidance for developing and evaluating complex interventions [13].

We have learned from the intervention components and characteristics associated with QoL improvements in cancer survivors [6]. Adjustment‐focused elements that apply across cancers can be supplemented with problem‐focused elements related to the target population [8]. Here, desired support encompassed several components of the PRISMS taxonomy (e.g., ‘Information about available resources’; ‘Information about the condition and its management’) [7].

Considering the TIDieR checklist [12] from the outset provided transparency around people with LGG's diverse preferences for the design, content, and delivery of a self‐management intervention. We found preference for blended online and face‐to‐face access to information and support, which aligns with preferences from a fatigue‐specific self‐management intervention for people with brain tumours [14]. To facilitate engagement, flexibility is required in how and when support is delivered likely due, in part, to the varied time needed to accept the incurable condition. Repeat ‘needs assessments’ could ensure information and support aligns with individual needs at different timepoints; assessments could be integrated within an online platform which directs users to relevant sections based on responses [15]. These findings relate to action one from Howell et al.’s Call to Action [9], to prepare patients and caregivers for active involvement in care.

A recent scoping review of rehabilitation interventions for people with brain tumours called for greater involvement of adults with brain tumours and caregivers in their development; noting the benefits of tailoring and need for interventions that consider people's work and social lives [16]. Hence, our involvement of multiple stakeholders in co‐design of a tailorable self‐management intervention that encompasses all aspects of day‐to‐day life is novel and timely. Involving multiple perspectives provided understanding of how to facilitate provider‐recipient relationships, which is crucial for guiding patient‐centred care in neuro‐oncology [17]. Overall, our findings align with Howell et al.’s action two, to support patients as partners in co‐creating self‐management support [9].

Key uncertainties were raised around (supporting) self‐management, particularly for people with cognitive and communication difficulties; for example, concerns around technology accessibility, such as navigating and processing digital information, which may require referrals to specialist HCPs (e.g., Clinical Neuropsychologist). These will need careful consideration when progressing to intervention co‐development. In addition, our findings raised concerns around staffing levels, HCP training, access to specialist professionals, and referral pathways; corroborated in a survey of HCPs' views on inequalities in access to neuro‐oncology supportive care [18]. Support providers may, therefore, require training to understand neuro‐oncology‐specific issues. We could learn from the effectiveness of an existing self‐management support training programme for cancer nurses, which improved trainees' confidence with 15 support skills, including establishing rapport, tailoring strategies, and developing action plans [19]. This aligns with Howell et al.’s action three, to prepare the workforce to facilitate effective self‐management [9].

### 4.1 Implications (Clinical and Research)

Future research will co‐develop, test, and evaluate our prototype intervention, considering the APEASE criteria (acceptability; practicability; effectiveness; affordability; side‐effects; equity) [20] to ensure potential for real‐world implementation. Evidence on cost‐effectiveness is critical for policy makers and service providers when making implementation decisions [8]; however, cost‐effectiveness for existing self‐management interventions is unclear [6]. Early insights from an ongoing economic evaluation in Ways Ahead suggest supporting self‐management could alleviate the toll of informal caregiving and offset costs related to easing the caregiving burden.

While a guiding principle of the intervention is to complement but not replace standard care, it is possible that the intervention may become the primary support mechanism for many people with LGG, due to inequalities in access to neuro‐oncology supportive care [18]. Inequalities may be exacerbated in people living rurally with limited access to social and practical support, those with weaker support networks, or those from communities that often have poorer healthcare experiences (e.g., minority ethnic groups). This has implications for both intervention development and implementation, for example, when considering modes of delivery, content, training requirements for those delivering the intervention, and safeguarding.

### 4.2 Limitations

Due to interview demands and possible self‐selection bias, Phase one participants may have comprised those with more interest and capacity to take part, potentially influencing the breadth of experiences represented. Phase two involved more people with cognitive impairment to ensure that intervention design fully considered people's abilities to access and navigate digital information. Still, cognitive impairments brought challenges with processing the information presented in co‐design activities. To help overcome this, we sought advice from a Speech and Language Therapist on appropriate communication methods and adapted group materials from written to visual information.

The main researcher (BR) involved in data collection and analysis had a non‐clinical health psychology background, which may have variably influenced the depth and interpretation of the collected data. However, the multidisciplinary project team had wide‐ranging research and clinical expertise, which ensured different perspectives were considered.

To minimise participant burden we did not collect information on ethnicity, though participants were mostly White British, which is a limitation. Recently, there has been increased focus on improving inclusion of underserved groups in healthcare research. Therefore, future intervention development should seek to involve minoritised populations from the outset. This could be addressed by including ethnicity within purposive sampling strata to ensure participant selection leads to diversity and inclusion of underserved groups.

## 5 Conclusion

This co‐design work builds upon systematic reviews [2, 6] and empirical findings from Ways Ahead to represent the early design of a prototype supported self‐management intervention for people with LGG. This advances understanding of the potential actions required to facilitate self‐management and improve QoL for people with LGG, with scope for wider applicability to other brain tumour groups.

## Author Contributions

L.S., P.G., R.B., J.L., S.W., V.A.S., T.F.: conceived the Ways Ahead study and secured funding. L.S., B.R: designed study. B.R., L.S., L.D.: data collection. B.R.: data coding and analysis. B.R.: wrote initial draft of paper. All authors: critically reviewed paper and approved final version for submission.

## Funding

This work was supported by funding from The Brain Tumour Charity (GN‐000435).

## Conflicts of Interest

The authors declare no conflicts of interest.

## Supporting information


Supporting Information S1

Supporting Information S2

## Data Availability

The data that support the findings of this study may be available from the Chief Investigator (Professor Linda Sharp; linda.sharp@ncl.ac.uk) upon reasonable request. The data are not publicly available due to privacy or ethical restrictions.

## Endnotes

^1^
Caregivers are distinct from ‘carers’ as they provide unpaid family support, rather than paid professional care.
