# Supplementary material for: Desired Support and Design Preferences for a Supported Self‐Management Intervention for People With Lower‐Grade Gliomas: Co‐Design Findings From the Ways Ahead Project
Source: Psychooncology. 2026 Jul 27;35(7):e70561. doi: 10.1002/pon.70561 (PMC13405862; doi:10.1002/pon.70561)
Supplement: Supplementary file 1 — Supporting Information S1 [file PON-35-e70561-s001.pdf]

Designing a support programme for people living with a brain tumour: your thoughts

Introduction

We are a research team at Newcastle University, funded by The Brain Tumour Charity.

Our project is looking at improving support for people living with a brain tumour. We are designing a support programme and would like your thoughts on this.

In this survey, we will introduce the ideas for the support programme and ask you some questions about these.

This survey will take 10 to 15 minutes to complete.

To navigate the survey, please select **'Next'** at the bottom of each page to progress, or select **'Previous'** if you need to go back a page.

If you accidentally leave the survey before finishing, you can restart by re-clicking the original link. Equally, if you wish to take a break, you can select **'Finish later'** at the bottom of the page. If you do this, please remember to come back and complete it.

If you have any questions or would like to provide additional feedback, the research team can be contacted on the details below:

Ben Rimmer and Linda Sharp
Email: waysahead@newcastle.ac.uk

What have we done so far?

We have spoken to people living with a brain tumour and their family-members. They have told us that living with a brain tumour can have many challenges, such as fatigue or impact on memory. They also told us that these challenges can impact on many areas of life including work, hobbies, social life, and overall quality of life.

There are many things that people can do that can help with these challenges, if they have the right information, resources, and support. This is called "self-management".

Self-management has been defined as "*awareness and active participation by the person in their recovery, recuperation, and rehabilitation to minimise the consequences of treatment, promote survival, health and well-being*".

In our project, we are developing a support programme that will help people living with a brain tumour to self-manage. We need your input and feedback to make sure that this support programme meets the needs of people living with a brain tumour.

What am I being asked to do?

On the following pages, we provide an outline of what the support programme might include and how it might be delivered. These are based on suggestions from people living with a brain tumour and their family-members. We would now like you to tell us what you think, to help us develop it further.

The support programme is targeted at people living with a brain tumour, but we also value the feedback of family-members/close friends, because they can have such an important role in supporting someone living with a brain tumour.

This survey has three sections:

1. Demographics - questions to learn a bit about you.
2. Programme design - questions to gather your views and opinions on what the support programme should look like.
3. Use of health services - questions to capture an overview of the support that is already being received.

There is space throughout, and again at the end, to give your thoughts and feedback on any issues that you think we have not covered.

Patient or family/friend

Are you someone living with a brain tumour or someone involved in the support of someone living with a brain tumour? ✱ *Required*

- Person living with a brain tumour
- Family-member/Close friend

Demographics - Patient

What is your age group?

- <30
- 30-39
- 40-49
- 50-59
- 60-69
- >70

What is your gender?

- Male
- Female
- Other
- Prefer not to say

If you selected Other, please tell us more:

What is your diagnosis?

If you selected Other, please tell us more:

Approximately, when did you receive your diagnosis? (you only need to tell us month and year)

Where do you usually get your information or support with living with a brain tumour? (select all that apply)

- ☐ Clinical team
- ☐ GP
- ☐ Support group(s)
- ☐ Online forum
- ☐ Web searches
- ☐ Charity websites
- ☐ Information booklets from the hospital
- ☐ Other

If you selected Support group(s), please tell us whether they are:

- ○ Face-to-face
- ○ Online
- ○ Both

If you selected Other, please tell us more:

Has a family-member or close friend been involved in supporting and helping you since your diagnosis?

- ○ Yes
- ○ No

How important is it for you that family-members/close friends have access to information about living with a brain tumour?

- ○ Very important
- ○ Somewhat important
- ○ Not very important
- ○ Not important at all

Demographics - Family/friend

What is your age group?

- <30
- 30-39
- 40-49
- 50-59
- 60-69
- >70

What is your gender?

- Male
- Female
- Other
- Prefer not to say

If you selected Other, please tell us more:

What is the diagnosis of the person you support?

If you selected Other, please tell us more:

Approximately, when did the person you support receive their diagnosis? (you only need to tell us month and year)

Where does the person you support usually get their information or support with living with a brain tumour? (select all that apply)

- ☐ Clinical team
- ☐ GP
- ☐ Support group(s)
- ☐ Online forum
- ☐ Web searches
- ☐ Charity websites
- ☐ Information booklets from the hospital
- ☐ Other

If you selected Support group(s), please tell us whether they are:

- ○ Face-to-face
- ○ Online
- ○ Both

If you selected Other, please tell us more:

Where do you usually get your information or support with supporting someone living with a brain tumour? (select all that apply)

- ☐ Clinical team
- ☐ GP
- ☐ Support group(s)
- ☐ Online forum
- ☐ Web searches
- ☐ Charity websites
- ☐ Information booklets from the hospital
- ☐ Other

If you selected Support group(s), please tell us whether they are:

- ○ Face-to-face
- ○ Online

○ Both

If you selected Other, please tell us more:

Programme design - Example support programme

The next section will cover **programme design** to get your feedback on what you think the support programme should look like.

Throughout this section, when we refer to 'support' we mean, for example, more opportunity to ask questions, or training to learn about more ways to manage.

Below, we present an example support programme that is still in development. We would like to know what you think about each part of its design.

Our example supported self-management programme

The programme will start at the end of initial treatment. It will ***NOT*** replace any standard follow-up appointments with the hospital team - it will be offered in addition to those.

Below, to help make things clear, we refer to the person living with a brain tumour as a "patient".

1. The patient is referred to the support programme from someone in their care team at the hospital.
2. The patient is offered an appointment - by phone or video-call - with a "brain tumour advisor". This person will be trained and have expertise in the needs of people living with a brain tumour. This appointment will identify anything that the patient needs support with, to help them make the "transition" back to daily life.
3. The advisor will provide the patient with log-in details for an interactive website. This will be able to be used on a computer, tablet, or phone. The advisor will introduce the patient to the bits of the website that they might find most helpful, based on what they said during their appointment.
   The website/web-app will remain available indefinitely and will include things like:

   - An information bank, covering important issues (e.g. seizures, transport) with advice on how to manage, and links to available support resources.
   - Facility to set reminders (e.g. to remember medications).
   - Interactive activities, including (1) a facility to set goals or make plans for everyday life and (2) "exercises" to help with emotions and mood.
   - A diary which can be used to record symptoms, hospital appointments, feelings or mood, etc.
   - Patient forum, with the facility to ask other patients questions, or offer advice to others.
   - Facility to ask someone with expertise in brain tumours a question.

4. Six-months later, the patient will be offered a follow-up appointment with the advisor. This will be by phone or video-call. It will be a "check-in" to see how the patient is doing. The patient will have the opportunity to talk about things they need support with or to ask questions. The website/web-app will still be available to the patient after this appointment.

Programme design - Content

Please think about the content of a support programme. What topics would you like to see included?

	Please select all that apply.	Please select the three most important topics.
Information about the tumour and potential consequences	☐	☐
Fatigue	☐	☐
Seizures	☐	☐
Headaches	☐	☐
Motor dysfunction	☐	☐
Memory	☐	☐
Speech, language, and communication	☐	☐
Concentration	☐	☐
Body image	☐	☐
Personality changes	☐	☐
Managing mood and emotions	☐	☐
Dealing with uncertainty about the future	☐	☐
Acceptance	☐	☐
Coping strategies	☐	☐
Relationships with family and other people	☐	☐
Maintaining independence	☐	☐
Hobbies	☐	☐
Work	☐	☐
Finances	☐	☐
Transport	☐	☐
Having a healthy lifestyle (e.g. being active, eating well)	☐	☐
Other	☐	☐

If you selected Other, please tell us more:

Do you think the support programme should include information or support for family-members and close friends of

people living with a brain tumour?

- Yes
- Maybe/not sure
- No, not essential

If yes, what topics/areas do you think should be included? (e.g. information about treatment side-effects or managing emotions)

Programme design - When

How important is it to you that the referral to this support programme comes from someone in the care team at the hospital?

- Very important
- Somewhat important
- Not very important
- Not important at all

If you would like to tell us more, please use the text box below:

We have suggested that initial referral to this support programme would come at the end of initial treatment. What do you think about this timing?

- About right
- Too early
- Too late

If you would like to tell us more, please use the text box below:

We have suggested a six month follow-up with an advisor. This appointment will be a "check-in" to give the patient an opportunity to talk about things they need support with or to ask questions. What do you think about this timing?

- About right
- Too early
- Too late

If you would like to tell us more, please use the text box below:

Do you think there needs to be another follow-up appointment with the advisor around 12 months? (i.e. 6 months after the last appointment with the advisor)

- Yes
- Maybe/not sure
- No, not needed

If you would like to tell us more, please use the text box below:

Do you think it would be important to be able to contact the advisor between appointments, if you had questions or needed support?

- Yes
- Maybe/not sure
- No

If you would like to tell us more, please use the text box below:

Do you think this support programme should be available to those who are already longer from diagnosis (e.g. more than 2 years)?

- Yes
- Maybe/not sure
- No

If you would like to tell us more, please use the text box below:

Programme design - Where and Who

Where

We have suggested that the follow-up appointments with the advisor will be provided as a video call or phone call. What would be your preferred method for this appointment?

- Face-to-face
- Video/Phone call
- Online chat
- Other

If you selected Face-to-face, please tell us where you would like the follow-up appointments to be located:

- Hospital
- Somewhere else

If you selected Somewhere else, please tell us more:

If you selected Other, please tell us more:

If you would like to add more information to support your answers, please use the text-box below:

Who

We have suggested that support would be provided by a trained "brain tumour advisor" who has a good

understanding of the needs of people living with a brain tumour. Would this be acceptable?

- Yes
- No, I would prefer someone from the care team at the hospital
- No, I would prefer someone else

If you would prefer support to be provided by someone from the care team at the hospital, who would you like that to be?

- Clinical nurse specialist
- Another health professional
- Don't mind/not sure

If you selected Another health professional, please tell us more:

If you would like to add more information to support your answers, please use the text-box below:

Programme design - How

We have suggested that information and support could be provided through an online interactive website/web-app. How confident would you feel in making use of this?

- ○ Very confident
- ○ Happy to give it a try
- ○ Would be OK to use it if I had some instructions, or if someone showed me how to use it
- ○ Not confident at all

How willing would you be to make use of an interactive website/web-app to find information and support?

- ○ I would definitely use it
- ○ I would probably use it
- ○ I probably would NOT use it
- ○ I definitely would NOT use it

Do you think the support programme should include something else in addition to the appointments with the advisor and the website/web-app?

- ○ Yes
- ○ No
- ○ Not sure

If yes, what else would you like to see included? (select all that apply)

- ☐ Online support group
- ☐ Face-to-face support group
- ☐ Information booklet
- ☐ Telephone support
- ☐ Other professional appointment
- ☐ Other

If you selected Other, please tell us more:

If you would like to add more information to support your answers, please use the text-box below:

Use of health services

The next section asks about appointments with professionals within and outside the NHS, in the last 12 months.

This will help us to understand what support is currently being used.

This section is about the **support received by people living with a brain tumour**. If you are supporting someone living with a brain tumour, please complete this section from their perspective (i.e. tell us about the appointments they have had).

If you can't remember, it is OK to give us your best guess.

Use of health services - within NHS

In the last 12 months, have you had any overnight stays in hospital related to the brain tumour?

- Yes
- No

Approximately how many nights in total have you stayed overnight in hospital in the last 12 months? (please state a number)

Thinking about the last 12 months, which of these professionals have you seen regarding the brain tumour? Please only include professionals you have seen within the NHS.

	Have you seen this professional?		If yes, how many appointments in the last 12 months? (please state a number)
	Yes	No	
Clinical nurse specialist			
Epilepsy nurse specialist			
Neuropsychologist			
Occupational therapist			
Oncologist			
Physiotherapist			
Speech and language therapist			
Surgeon			
Someone else (1)			
Someone else (2)			

If you selected Someone else, please tell us more:

Thinking about the last 12 months, which of these professionals have you seen/spoken to in your GP practice regarding the brain tumour?

	Have you seen/spoken to this professional?			
	Yes	No	If yes, how many face-to-face appointments in the last 12 months? (please state a number)	If yes, how many phone call appointments in the last 12 months? (please state a number)
GP (family doctor)	○	○		
Nurse in your GP practice	○	○		

If you would like to add more information to support your answers, please use the text-box below:

Use of health services - outside NHS

Please now think about support and appointments with professionals outside the NHS. In the last 12 months, which of these professionals have you seen for things related to the brain tumour?

	Have you seen this professional?		If yes, how many appointments in the last 12 months? (please state a number)
	Yes	No	
Counsellor	○	○	
Occupational therapist	○	○	
Physiotherapist	○	○	
Psychologist	○	○	
Speech and language therapist	○	○	
Benefits advisor	○	○	
Someone else (1)	○	○	
Someone else (2)	○	○	

If you selected Someone else, please tell us more:

If you would like to add more information to support your answers, please use the text-box below:

Final comments

Thank you very much for answering our questions, your feedback is really appreciated. If you have any final comments, please use the text-box below:

Thank you

Thank you for completing our survey.

When we have finished this part of the study, we will upload a summary of findings to the project website: [Ways Ahead | Ways Ahead | Newcastle University (ncl.ac.uk)]

We will also ask the Brain Tumour Charity to share our findings so that you can see how we have responded to your feedback. This means that we don't need to ask for, and store, your personal details.

If you have any questions or would like to provide further feedback, you can contact the lead researcher, Ben Rimmer, on: waysahead@newcastle.ac.uk

---

Key for selection options

4 - What is your diagnosis?

Grade II astrocytoma
Grade III astrocytoma
Grade II oligodendroglioma
Grade III oligodendroglioma
Acoustic neuroma
CNS lymphoma
Glioblastoma
Haemangioblastoma
Meningioma
Pituitary adenoma
Unspecified glioma
Don't know
Other

10 - What is the diagnosis of the person you support?

Grade II astrocytoma
Grade III astrocytoma
Grade II oligodendroglioma
Grade III oligodendroglioma
Acoustic neuroma
CNS lymphoma
Glioblastoma
Haemangioblastoma
Meningioma
Pituitary adenoma
Unspecified glioma
Don't know
Other
