# Supplementary material for: Desired Support and Design Preferences for a Supported Self‐Management Intervention for People With Lower‐Grade Gliomas: Co‐Design Findings From the Ways Ahead Project
Source: Psychooncology. 2026 Jul 27;35(7):e70561. doi: 10.1002/pon.70561 (PMC13405862; doi:10.1002/pon.70561)
Supplement: Supplementary file 2 — Supporting Information S2 [file PON-35-e70561-s002.docx]

**Desired support and design preferences for a supported self-management intervention for people with lower-grade gliomas: co-design findings from the Ways Ahead project**

Ben Rimmer^1*^, Sophie Williams^2^, Joanne Lewis^2^, Lizzie Dutton^1^, Richéal Burns^3,4^, Pamela Gallagher^5^, Vera Araújo-Soares^1,6^, Tracy Finch^7^, Linda Sharp^1^

Author affiliations:

1. *Population Health Sciences Institute, Newcastle University, Newcastle University Centre for Cancer, Newcastle upon Tyne, England*
2. *Newcastle upon Tyne Hospitals NHS Foundation Trust, Newcastle upon Tyne, England*
3. *Faculty of Science, Atlantic Technological University, Sligo, Ireland*
4. *Health and Biomedical Strategic Research Centre, Atlantic Technological University, Ireland*
5. *School of Psychology, Dublin City University, Dublin, Ireland*
6. *Centre for Preventive Medicine and Digital Health, Department for Prevention of Cardiovascular and Metabolic Disease, Medical Faculty Mannheim, Heidelberg University, Heidelberg, Germany*
7. *Department of Nursing, Midwifery and Health, Northumbria University, Newcastle upon Tyne, England*

**Corresponding author: Ben Rimmer, Population Health Sciences Institute, Newcastle University, Ridley Building 1, Newcastle upon Tyne, NE1 7RU.
Email: ben.rimmer@newcastle.ac.uk; Phone: 0044 (0)7704 300 509*

Contents

[Supplementary table 1. Supporting publications from the Ways Ahead project indicating evidence-base for the intervention 2](#_Toc232495798)

[Supplementary table 2. Co-design activities 4](#_Toc232495799)

[Supplementary table 3. Design preferences for a supported self-management intervention 6](#_Toc232495800)

[Supplementary table 4. Early prototype of a supported self-management intervention 13](#_Toc232495801)

## Supplementary table 1. Supporting publications from the Ways Ahead project indicating evidence-base for the intervention

| **Ways Ahead publication** | **Brief summary** |
| --- | --- |
| Rimmer, B., Dutton, L., Lewis, J., Burns, R., Gallagher, P., Williams, S., ... & Sharp, L. (2020). Ways Ahead: developing a supported self-management programme for people living with low-and intermediate-grade gliomas-a protocol for a multi-method study. BMJ open, 10(7), e041465. | Protocol with full details of the methodology for the Ways Ahead project, including project stages, setting, eligibility criteria, recruitment, data collection, patient and public involvement, and ethical considerations. This paper details how the methodology was tweaked for each interview set (people with LGG, informal caregivers, healthcare professionals). |
| Rimmer, B., Balla, M., Dutton, L., Williams, S., Lewis, J., Gallagher, P., ... & Sharp, L. (2024). “It changes everything”: Understanding how people experience the impact of living with a lower-grade glioma. Neuro-Oncology Practice, 11(3), 255-265 | Qualitative analysis of the people with LGG interview data. This paper highlights the everyday impacts and supportive care needs of people with LGG. These findings reflect participants’ experiences with symptoms (e.g. fatigue, seizures) and impairments (e.g. motor dysfunction, cognitive deficits), and how these, in turn, drive impacts on daily living (including on work, relationships, social activities, and transport). |
| Rimmer, B., Balla, M., Dutton, L., Lewis, J., Brown, M. C., Burns, R., ... & Sharp, L. (2023). Identifying and understanding how people living with a lower-grade glioma engage in self-management. Journal of Cancer Survivorship, 18(1), 1837–1850. | Qualitative analysis of the people with LGG interview data. This paper comprehensively highlights how people with LGG engage in self-management, through 123 self-management strategies (e.g. receiving support from family; accepting the tumour and its consequences) across 20 self-management strategy types (e.g. using support; meaning making). |
| Rimmer, B., Balla, M., Dutton, L., Williams, S., Araújo-Soares, V., Gallagher, P., ... & Sharp, L. (2024). Barriers and facilitators to self-management in people living with a lower-grade glioma. Journal of Cancer Survivorship, 1-14. | Qualitative analysis of the people with LGG interview data. This paper highlights how people with LGG distinctively experience wide-ranging factors influencing their ability to self-manage. This includes multiple, often co-occurring challenges with 18 factors (e.g. cognitive functioning) across five categories (e.g. health status), primarily with knowledge and acceptance of their incurable condition, impact of seizures and cognitive deficits, and access to (in)formal support. |
| Walker, H., Rimmer, B., Dutton, L., Finch, T., Gallagher, P., Lewis, J., ... & Sharp, L. (2023). Experiences of work for people living with a grade 2/3 oligodendroglioma: a qualitative analysis within the Ways Ahead study. BMJ open, 13(9), e074151. | Qualitative analysis of the people with LGG interview data. This paper highlights the work experiences of people with LGG, including how symptoms and impairments, work environment, and employer support influence work experiences and have implications for the importance of work in rehabilitation for people with LGG. |
| Rimmer, B., Finch, T., Balla, M., Dutton, L., Williams, S., Lewis, J., … & Sharp, L. (2024). Understanding supported self-management for people living with a lower-grade glioma: implementation considerations through the lens of normalisation process theory. Health Expect. 27(3), e14073. | Qualitative analysis of the healthcare professional and people with LGG interview data. This paper highlights the collective nature of, and provides insight into the individual roles within, supported self-management. Key implementation considerations include ensuring awareness of, and access to, support; building strong HCP-support recipient relationships; and careful inclusion of close family and friends. There are pertinent challenges with identifying support needs, resistance to support, training for healthcare professionals, and healthcare professional cooperation. |
| Rimmer, B., Balla, M., Dutton, L., Burns, R., Araújo-Soares, V., Finch, T., … & Sharp, L. (2024). “It’s a delicate dance” How informal caregivers experience the role and responsibilities of supporting someone living with a lower-grade glioma. Neuro-Oncology Practice, 12(2), 340-350. | Qualitative analysis of the caregiver interview data. This paper highlights the wide-ranging role and responsibilities of being a caregiver for people with LGG, including providing cognitive, emotional, and practical support, and helping to navigate the healthcare system. This also outlines multiple challenges with fulfilling the caring role, including conflict with work/childcare and trying not to limit the care recipient’s independence. |
| Rimmer, B., Balla, M., Dutton, L., Lewis, J., Burns, R., Gallagher, P., ... & Sharp, L. (2024). ‘A constant black cloud’: the emotional impact of informal caregiving for someone with a lower-grade glioma. Qualitative Health Research, 34(3), 227-238. | Qualitative analysis of the caregiver interview data. This paper highlights the wide-ranging emotional responses to and impacts of the illness, uncertain prognosis, care recipient changes, and the toll of caregiving, for caregivers of people with LGG. This has implications for the emotional support needs of family/friends involved in supporting people with LGG. |
| Murrell, A. J., Rimmer, B., Dutton, L., Lewis, J., Burns, R., Gallagher, P., ... & Sharp, L. (2023). The nature and quality of support from informal networks for informal caregivers of low-grade glioma patients: A qualitative analysis within the Ways Ahead study. European Journal of Cancer Care, 2023(1), 4149412. | Qualitative analysis of the caregiver interview data. This paper highlights the wide-ranging emotional, instrumental, information, and appraisal support that caregivers may receive from strong/familiar (e.g. close friends) and weaker/unfamiliar (other caregivers) ties, to protect their wellbeing. This highlights the importance and value of extended networks. |

## Supplementary table 2. Co-design activities

| **Activity^a^** | **Participants** | **Characteristics** | **Intervention-related content covered** |
| --- | --- | --- | --- |
| **Phase one** | | | |
| *People with LGG interviews* (remote) | 28 | Female (n=12), Male (n=16); Grade 2 oligodendroglioma (n=10), Grade 3 oligodendroglioma (n=9), Grade 2 astrocytoma (n=9) | Desired support; experiences of support; intervention design preferences (e.g. what, when, and how). |
| *Caregiver interviews* (remote) | 19 | Female spouse (n=10), Male spouse (n=5), Mother (n=2), Sister (n=2) | Desired support (for interviewee and care recipient); experiences of support (and role in providing support); intervention design preferences. |
| *HCP interviews* (remote) | 25 | Clinical nurse specialist (n=6), Occupational therapist (n=4), Neuropsychologist (n=3), Physiotherapist (n=2), Clinical oncologist (n=2), Specialist allied health professional (n=2), Neurosurgeon (n=1), Neuroradiologist (n=1), Neurooncology support sister (n=1), Speech and language therapist (n=1), Epilepsy nurse specialist (n=1), Macmillan centre manager (n=1) | Available support; how support could be improved; feelings about, and role in, supporting self-management; intervention feasibility. |
| **Phase two** | | | |
| *People with brain tumours group A* (in-person) | 7 | Grade 1 Meningioma (n=4), Grade 2 Central Neurocytoma (n=1), Grade 4 Glioblastoma Multiforme (n=1), Grade 3 Ependymoma (n=1) | Main areas that people need/desire support with; what is required for effective self-management; intervention delivery modes (e.g. website, groups, face-to-face); accessibility (e.g. technology literacy; communication impairments). |
| *HCP group* (remote) | 6 | Neuropsychologist (n=1), Clinical oncologist (n=1), Clinical nurse specialist (n=1), Occupational therapist (n=1), Physiotherapist (n=2) | Ensuring complementarity to existing support; design, feasibility, and acceptability of early intervention prototype; integration of, and support for, caregivers; overcoming resistance to support. |
| *Caregiver group* (remote) | 4 | Female spouse (n=2), Male spouse (n=2) | Main areas that people need support with; desired integration of, and support for, caregivers; design, feasibility, and acceptability of early intervention prototype. |
| *People with brain tumours group B* (in-person) | 10^b^ | Grade 1 Meningioma (n=3), Grade 4 Glioblastoma Multiforme (n=2), Grade 3 Ependymoma (n=1), Grade 2 Astrocytoma (n=1) | Design, feasibility, and acceptability of early intervention prototype; desired integration of, and support for, caregivers. |
| *Intervention design survey* (remote) | 33 | People with brain tumours (n=21): Grade 2 astrocytoma (n=4), Grade 3 astrocytoma (n=2), Grade 2 oligodendroglioma (n=4), Grade 3 oligodendroglioma (n=1), Acoustic neuroma (n=4), Glioblastoma (n=3), Meningioma (n=3); Caregivers (n=12): Caregivers of Grade 2 astrocytoma (n=5), Grade 2 oligodendroglioma (n=2), Grade 3 oligodendroglioma (n=2), Glioblastoma (n=3) | Reactions to, feedback on, and acceptability of suggested intervention characteristics (e.g. how, who, when, where, what). |
| *HCP = Healthcare professional; LGG = Lower-grade glioma*  ^a^Timeline of co-design activities – phase one: three interview sets conducted simultaneously; phase two: survey data collection simultaneous with discussion groups (group order as presented).  ^b^Including three caregivers. | | | |

## Supplementary table 3. Design preferences for a supported self-management intervention

| **Intervention characteristic** | **Key findings and where these arose** | **Illustrative quotes from Phase one interviews^a^** |
| --- | --- | --- |
| *What (content)* | *People with LGG interviews:* More information about the condition and its treatment to know what to expect, in turn increasing awareness of what to seek support for. Topics covered need to encompass all symptoms, impairments, social and role implications as support needs will vary on an individual basis. Caregivers have their own support needs and need to understand what is going on to be able to support the person with LGG.  *Caregiver interviews:* Information on what to expect and how to manage symptoms and impairments; participants felt their role in helping to support engagement in self-management should be acknowledged  *HCP interviews:* Self-management support should incorporate problem-focused elements that encapsulate people with brain tumours’ wide-ranging support needs; encompassing physical, cognitive, psychological, emotional, as well as practical, social and role (e.g. work, finances).  *People with brain tumours groups A and B:* Support needed for memory deficits and psychological adjustment to help with acceptance, and find independence and control over one’s life. Suggested an extra support topic of ‘helping children to manage’.  *HCP group:* Need to outline what to expect at different points in the illness pathway, highlighting what might change or have a long lasting impact. People with brain tumours may have good performance status, but the emotional impact can be overwhelming, so need support with psychological adjustment, social and role implications. Support/training needed for caregivers to help them understand how they can look after themselves, communicate to children, and help implement self-management strategies in the home environment.  *Caregiver group:* Suggested that extra topics need to be considered, including: speaking with children, starting a family, seeking financial benefits.  *Survey:* The support topics selected as most important included: information about tumour and potential consequences, memory, managing mood and emotions, dealing with uncertainty about the future, maintaining independence, fatigue, seizures. Most respondents thought an intervention should include information and support for caregivers (e.g. knowing what to expect from the tumour and its treatment). | - “Not everybody knows that, if you have a brain tumour or seizure, whether you’ve got to hand your licence in. It’s something like that and guiding them right. You want to go onto like a three-year licence and stuff like that. You only find these things out when it happens.” – Pa31 (Male, Grade 2 oligodendroglioma) - “As well as the person that is physically impacted by the diagnosis as well, it's the nearest and dearest, family and friends, wives, husbands, partners, etcetera, and the support they might need.” – Pa15 (Male, Grade 2 astrocytoma) - “The carers and the people who are supporting people – the service users and the patients – need to be involved in a lot of this as well, because they are the people keeping things going and taking over.” – C01 (Female spouse) - “Help the [support recipient] to understand why they feel frustrated or why they’ve got no patience anymore and then ways to cope with that and self-manage. That to me is the essence of self-management, giving people not just the understanding but the tools to do it for themselves.” – C23 (Male spouse) - “There’s the lifestyle type, support with occupation, finance, psychological, and physical things. So I think even dietary, health and fitness, even things like people going away places, getting information about that, insurance company listings. So I suppose it’s having access to all these sort of things.” – HCP39 (Clinical nurse specialist) - “Short information videos about specific problems, perhaps how other people have dealt with things as well, other patients, patient stories.” – HCP43 (Specialist allied health professional) |
| *What (materials)* | *People with LGG interviews:* A central information resource with contact details, advice on what to expect, how to self-manage, and signposting to available support. Important to keep information up to date to ensure signposted support is still available. Individual preferences for an online resource or a physical leaflet, with consideration of technology literacy and level of detail.  *Caregiver interviews:* Contact details sheet of relevant HCPs alongside an information helpline and ‘toolkit’ as a central information resource specific to brain tumours, to signpost to available support. Need to consider technology literacy, level of detail, visual information etc. when determining the feasibility of the support recipient engaging with presented information.  *HCP interviews:* Look at the potential to adapt and incorporate existing support. Focus on advice and signposting, through the platform of an information ‘toolkit’, to ensure and maintain awareness of available support. Make sure that information is presented in an accessible manner (e.g. level of detail, visual information, font size).  *People with brain tumours groups A and B:* Information on self-management strategies would be beneficial, presented in an option of audio, video with picture, and written online, or physical booklet; individual preference influenced by extent of cognitive impairment. Information should also be available to caregivers as they are important in supporting self-management.  *HCP group:* Equip people with brain tumours with the skills and information they need to have the best chance of thriving. Information resources are a crowded space, there is a need to create a standardised central resource that brings resources together.  *Caregiver group:* Videos were the preferable format of delivering information and education; they need to be short and snappy or delivered in parts, if they need to be longer. Also post practical advice from other people with brain tumours to share strategies that have been helpful to self-manage; allows access to a ‘bank’ of tried and tested strategies to try for themselves.  *Survey:* Most respondents were confident and willing to use a web-app to access information and support. | - “I think a central depository of information… so if I want to know stuff about diet, somewhere I can call or I can look up.” – Pa13 (Male, Grade 3 oligodendroglioma) - “It's ask, ask, ask but it would be good to know that actually, I'm asking for something that I know is there or I am eligible for. So, I know that it exists in the first place rather than having to ask whether it exists in the first place.” – Pa15 (Male, Grade 2 astrocytoma) - “Macmillan give you leaflets and I guess they’re trying to be helpful but most things are very general. What would be nice would be to have an internet access thing, a toolkit online where all his details are already there and I only get given links to things which are appropriate to him.” – C07 (Female spouse) - “To have someone pick up the phone and go, “Right. This is what’s happening. This is where I am. This is the situation I’m in, I need help with X, Y, and Z, where do I go?”” – C06 (Sister) - “Making it accessible in different ways, so maybe in paper format or in videos. We found that people are quite liking the education sessions that are pre-recorded so just being able to get the information in different ways so that they can access it differently if they want, whether it be sent out in a leaflet or an online video.” – HCP52 (Epilepsy nurse specialist) - “Having something that I could direct patients to that’s easy, that holds a whole lot of information that would cover most of the main areas, do you know what it would do for me, it would ease my conscience.” – HCP49 (Occupational therapist) |
| *Who (if not self-administered)* | *People with LGG interviews:* Preference for hearing about potential challenges and how to self-manage from people who had experience of the condition; more important that the person was knowledgeable and sympathetic to the condition, than whether they were a HCP or ‘patient champion’. The availability of both would be optimal.  *HCP interviews:* Where a ‘support lead’ is required, this person must be knowledgeable and sensitive to the needs of people living with a brain tumour, but this could be an ‘expert patient’ or a HCP.  *People with brain tumours groups A and B:* Support groups should be run by ‘patient champions’, as other people with brain tumours are able to comprehend their needs; HCP input is still valuable for signposting and access to available support. Would contact clinical team before an advisor for anything symptom-related, due to perceived expertise; though would contact an advisor for practical advice so they do not feel they are using clinical time.  *HCP group:* An advisor or HCP would be helpful to signpost the person with brain tumour to appropriate support at the appropriate time; though some sites lack specialist HCPs, contributing to long waiting lists. Referral to an intervention from a HCP legitimises the reputability of an information source.  *Survey:* Most respondents indicated that it would be acceptable for support to be provided by a trained ‘brain tumour advisor’ who has a good understanding of the needs of people living with a brain tumour. Most respondents indicated that it is at least somewhat important to be referred to an intervention by someone in the care team at the hospital. | - “To have a health professional and somebody who’s been through this who can answer things clearly or say, “Do you know what? I don’t know the answer to that but I know somebody who’s been through what you’re going through”” – Pa18 (Female, Grade 3 oligodendroglioma) - “Lay people or professionals, a mix, as long as they’re competent, they know what they’re talking about and they’re empathetic.” – Pa16 (Male, Grade 3 oligodendroglioma) - “Someone taking charge who is knowledgeable and in a position of trust from the patient’s perspective.” – HCP28 (Clinical oncologist) |
| *How* | *People with LGG interviews:* Groups can be valuable for education and support, facilitating the opportunity to connect, and share advice and experiences with ‘similar others’. However, there were concerns about meeting people in a worse situation or who may deteriorate, hindering the inclination to join a group. Face-to-face groups were seen as beneficial, though challenging due to timing and transport, so online was deemed more feasible.  *Caregiver interviews:* Desired the opportunity for caregivers and people with LGGs to engage with similar others to share advice and experiences. A range of options to interact with others are needed, as groups are not for everyone.  *HCP interviews:* Considered the value of education courses on how to self-manage and groups for support, though acknowledged that one size will not fit all, so a blended approach that gives people an option of how to access information may be optimal. Integrating caregivers in the delivery of support is important, though engagement in self-management should not be reliant on a strong support network.  *People with brain tumours groups A and B:* Support groups (online or face-to-face) afford the opportunity to share practical advice and emotions with ‘similar others’ to reduce loneliness; face-to-face is preferred for a more ‘relaxed’ atmosphere and to avoid challenges with technology literacy. Appointments with an advisor should be similar in structure to a holistic needs assessment to comprehensively capture the individual’s needs.  *HCP group:* Originality in an education course (e.g. training resilience), though challenge with potential attendance levels; blended approach of online/face-to-face may allow people to access the same information in a way that is acceptable to them.  *Caregiver group:* Ensure, where possible, that family have access to information and education as they have a critical role in implementing this in the home environment and often assume the responsibility of information seeking and arranging support.  *Survey:* Several respondents suggested a blended approach of a web-app and support group would be beneficial. | - “I go on the brain tumour support groups. So I use that for a lot of information. Plus you can give advice to other people and people can give advice to you of their experiences and that which is quite a good help sometimes.” – Pa30 (Male, Grade 3 oligodendroglioma) - “The downside to it, obviously, I’ve seen a lot of people not make longer-term. You know, you make friends in support groups and stuff and they get ill and they just don’t make it, you know.” – Pa17 (Female, Grade 3 oligodendroglioma) - “The online option is very useful in terms of ease and it not feeling like it’s eating up your day but obviously there are positives to meeting people face to face so yes, I think mixed views.” – Pa40 (Female, Grade 2 astrocytoma) - “There’s always self-help, there’s always understanding from other carers that you can be in touch with to find out how they managed that situation.” – C08 (Mother) - “If you really understand about the process of self-managing yourself because somebody's taken you through that and taught you how to self-manage, then you've got more ability to do it. So, I think an individual course, teaching somebody how to self-manage would be beneficial.” – HCP21 (Physiotherapist) - “Immediately I thought, “Families, carers need to be part of this as well,” because I think they can reiterate back to patients what the advice has been or what they’ve actually learnt.” – HCP49 (Occupational therapist) |
| *Where* | *People with LGG interviews:* For face-to-face support, clinical settings should be avoided; prefer community-based locations with plenty of space, that don’t require too much reliance on transport. Cautious as to whether the same level of support from others could be achieved online.  *Caregiver interviews:* Online support was preferred as people with LGG can have issues with mobility or transport.  *HCP interviews:* Avoid a clinical setting, instead have local, community-based support, where possible. Alternatively, online, with a video platform, for example, may be more accessible.  *People with brain tumours groups A and B:* Face-to-face is preferred over online support groups, though there was no specificity on the location.  *HCP group:* May not get interaction with similar others in an online support group format; we are social beings that need connection to build resilience. The challenge of transport needs to be considered when determining the location of face-to-face support.  *Survey:* Most respondents suggested that it would be preferable for follow-up appointments with an advisor to be a phone/video call. | - “If people have got issues with driving that could be a difficulty as well. So, yeah, I guess [face-to-face support] would be good but there might be some practical barriers to overcome.” – Pa10 (Female, Grade 2 oligodendroglioma) - “I don’t know that you could easily do the same thing online, really. When people can get back to gathering and having live gatherings and meetings and things, you can’t beat that, really.” – Pa14 (Male, Grade 2 oligodendroglioma) - “An online support thing would be good, especially with things the way they are, and even because a lot of people do have mobility issues afterwards.” – C05 (Female spouse) - “There wouldn’t be a reason why you couldn’t have an alternative online. It would be possible to have the exact course done in a virtual setting if people preferred that.” – HCP28 (Clinical oncologist) - “To have something which can cover every area, so that it’s very local for people.” – HCP33 (Clinical nurse specialist) |
| *When* | *People with LGG interviews:* Certain information (e.g. about the condition and its treatment) is desired following diagnosis; information about self-management of symptoms and impairments, and psychological adjustment is desired around the six month timepoint, though needs change over time, so it would be beneficial for information to be readily available when the time comes. Once treatment is completed there is a feeling of isolation, as direct care reduces.  *Caregiver interviews:* Keep information brief and appropriate to the most important topics initially following diagnosis, when there is a lot on their mind, then broaden to additional topics further down the line, with needs changing over time.  *HCP interviews:* Most appropriate to have an intervention available following treatment, as people need time to adjust and realise what their support needs might be. People may have different needs at different times, so information should be accessible whenever is suitable for them.  *People with brain tumours groups A and B:* Information should be delivered immediately post-treatment, though certain advice (e.g. finances) would be beneficial immediately following diagnosis. Need the availability to contact someone ad-hoc as and when needs arise.  *HCP group:* Referral to an intervention should come at least six months in, as reality bites around 6-12 month mark; people already further from diagnosis (e.g. >2 years) should still be referred.  *Caregiver group:* Suggested it would be good to be able to self-refer into the programme at a time that is suitable for the person with LGG. Three 2-monthly follow-ups would be preferable for the first six months, with additional follow-ups at 12 and 18 months, or a time at the discretion of the person with LGG.  *Survey:* Many respondents thought referral to an intervention at the end of initial treatment was about right; some thought this was too late and should come at diagnosis. Most respondents indicated that an intervention should be available to those already longer from diagnosis (e.g. >2 years). Many respondents thought that follow-up appointments with an advisor six and 12 months since referral to an intervention would be beneficial; most respondents would like to be able to contact an advisor between appointments, should they have questions or need support. | - “After treatment is really important because you have your treatments and then you’re, not left on your own, but you see a lot of doctors and a lot of nurses and then all of a sudden there’s nothing. Maybe I just missed it but just a bit more interaction would be better.” – Pa26 (Female, Grade 2 oligodendroglioma) - “A support package put in place for you, and you can dip in and out of that at different times. For me, because I’m sort of a relatively structured person, that would be quite good.” – Pa11 (Male, Grade 2 oligodendroglioma) - “You’re living with this condition and as time goes on, it’s probably happened very subtly but we might have changed from how we were before or what we might need and different people’s conditions fluctuate.” – C12 (Female spouse) - “In those early days when you’ve got so much else to be thinking about, just a short check sheet or signposting would have been, I think, one of the most helpful things, the practical side of stuff. It would be marvellous if there was something that looked at what support do you need on that day one of diagnosis, the lead up to operation, post-operative, post-treatment, returning back to your new normal life.” – C02 (Female spouse) - “For most people who go through treatment, I think it’s busy, busy, busy, there’s something to work towards for months and months and then all of a sudden it’s, “Right okay,” and then often that’s where I find people tend to unravel a bit.” – HCP18 (Psychologist) - “The timing of it is trying to keep that on the radar, so that patients can decide at a different stage of their journey that they want to opt into support.” – HCP37 (Neuropsychologist) |
| *Tailoring* | *People with LGG interviews:* Everyone is individual with individual support needs, so it is difficult to present general information. Presenting information on everything can cause information overload, so people with LGG should be signposted to information that is simplified and relevant to their needs.  *Caregiver interviews:* People with LGG need to only access the information that is applicable to their needs to avoid information overload.  *HCP interviews:* Identify individual needs through a screening tool, then develop goals with input from people with brain tumours on what they want to achieve, acknowledging that everyone is different.  *People with brain tumours groups A and B:* Enable people with brain tumours to access information in a means that is acceptable to them, as communication impairment, for example, may precede the desire to participate in a group. Every person with a brain tumour may be too different to help each other directly with shared advice, so it is important that there is a tailored output following any needs assessment.  *HCP group:* Start with an individual, standardised needs assessment to identify the support needs of each person with a brain tumour and help them prioritise which elements of an intervention they need to engage with to avoid information overload. People respond to individual attention, so likely to be more successful if people with brain tumours feel an intervention is targeted. | - “Treatment should be designed around the individual, because everyone has got different problems. My problems are based around my disability. I need physical support… some people I know have emotional needs, but others don’t… you’ve got to design it around the individual.” – Pa13 (Male, Grade 3 oligodendroglioma) - “The only thing I’d really change is probably how much information people are given at the outset of their journey. It needs to be broken down.” – Pa18 (Female, Grade 3 oligodendroglioma) - “It’s like your front page that’s saying work, home, relationships, practical help, travel, I don’t know. Then click on that and you go into the next level of detail or support.” – C02 (Female spouse) - “It's identification of the issues and then it's developing with the patient goals of what they want to try and achieve and how we can support that through that and how they can support themselves.” – HCP21 (Physiotherapist) - “You need a screening tool, so the patient can identify the issues that are going to be pertinent to them, and then target the self-management.” – HCP29 (Clinical oncologist) |
| *HCP = Healthcare professional; LGG = Lower-grade gliomas*  ^a^Groups were not audio-recorded, but instead, the researchers took detailed notes. | | |

##
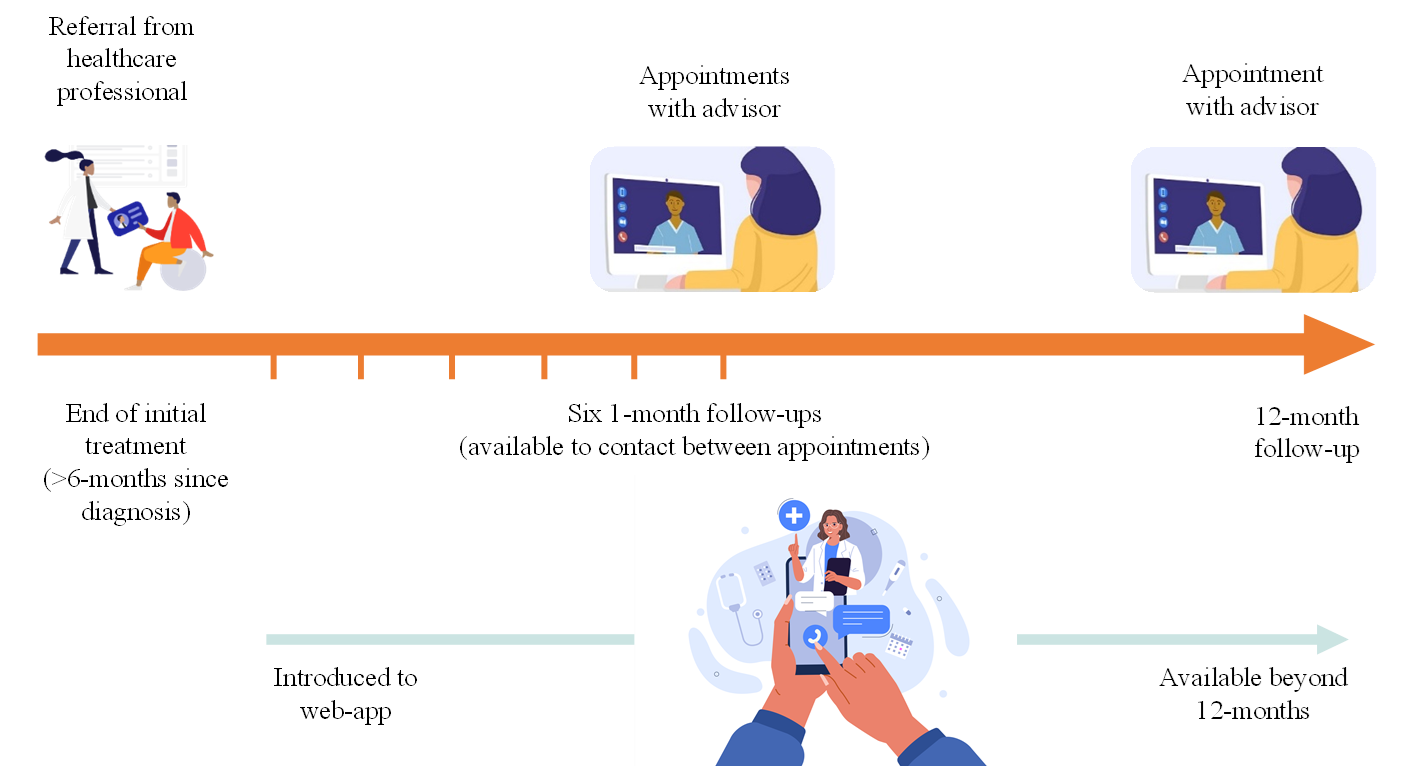
Supplementary table 4. Early prototype of a supported self-management intervention

| **Guiding principles of intervention** | 1. It will complement but ***not*** replace any standard follow-up care that is currently offered 2. It has to be feasible and implementable within NHS care pathways 3. There will be an option for informal caregiver (family/close friend) involvement at the patient’s discretion 4. It will seek to activate patient self-management within the rehabilitation stage 5. Support will be tailored where possible but still coherent with the aims of patient activation |
| --- | --- |
| **Intervention characteristic** | **Context** |
| *What (advisor appointments)* | - First advisor appointment: get to know the patient; find out what is important to them (desired topics, support needs), gauge the desire for involvement of an informal caregiver   - Introduced to web-app during appointment; signposted to most beneficial/relevant topics within the web-app, identified following needs assessment - Remaining advisor appointments: opportunity to re-evaluate support needs; act as a ‘check-in’ to gauge engagement in self-management; assess challenges to self-management |
| *What (web-app)* | - Information and support on different topics, including but not limited to: tumour and potential consequences, cognitive impairments, managing mood and emotions, dealing with future uncertainty, maintaining independence, managing fatigue, managing seizures   - Each topic will include an overview of what to expect and possible self-management strategies   - Information will be presented concisely with visual elements (e.g. short videos, patient stories, advice from other patients/healthcare professionals, evidence-based strategies) - The web-app will incorporate behaviour change techniques, such as interactive tools to facilitate self-management (e.g. goal setting, action plans, setting reminders for medication/healthcare appointments) - Space to record contact details of healthcare professionals within the patient’s care team; supplemented by a “This is what you can ask me” element that details what each healthcare professional can do for the patient   - Guidance on “How to make the most of your healthcare appointment” with the facility to plan questions to be asked in a consultation |
| *Who* | - Referral from a healthcare professional within the patient’s care team to legitimise the trustworthiness of the intervention - The advisor will be knowledgeable of, and sensitive to, the support needs of people with brain tumours; this could be a member of the Cancer Alliance, someone specifically employed to deliver the intervention, or a Clinical Nurse Specialist - The advisor will receive a training package, developed by the study team, including: evidence underpinning the intervention; detailed overview of the intervention and its components; an appointment guide (procedure, key discussion points, potential questions) - The web-app will be self-administered following the initial appointment |
| *How* | - Each advisor appointment will be held remotely (via video/phone call), per the patient’s preference - The advisor will be contactable between appointments via phone call, where necessary; this will not be a 24-hour service, so available contact hours will be advised - The web-app will be accessible online using a mobile, tablet, or computer device. Patients with issues with technology literacy/accessibility will be offered the possibility to request a concise physical copy of the information |
| *When and  how much* | - Referral will come at the end of initial treatment (and >6-months since diagnosis) once the patient has had time to recognise the effects of their diagnosis. Patients already post-treatment (and >6-months since diagnosis) can still be referred to the intervention - From point of referral, patients will be offered six 1-month follow-up advisor appointments, followed by a 12-month follow-up appointment (6-months after the previous appointment) - Patients will be encouraged to use the web-app as often as is helpful; it will remain available beyond the 12-month follow-up |
| *Tailoring* | - Each patient will have the opportunity to reflect on what is important to them; this will help tailor the guidance within advisor appointments. This can be re-evaluated in subsequent sessions, appreciating that support needs can change over time - There will be core topics, coherent with the aim of activating a patient’s self-management (e.g. managing the uncertainty of living with an incurable condition) - At the patient’s discretion, there will be capacity for informal caregiver involvement in the intervention (e.g. allowing attendance to advisor appointments; granting access to the web-app) |
